# The Ambiguous Base-Pairing and High Substrate Efficiency of T-705 (Favipiravir) Ribofuranosyl 5′-Triphosphate towards Influenza A Virus Polymerase

**DOI:** 10.1371/journal.pone.0068347

**Published:** 2013-07-10

**Authors:** Zhinan Jin, Lucas K. Smith, Vivek K. Rajwanshi, Baek Kim, Jerome Deval

**Affiliations:** 1 Alios BioPharma, Inc., South San Francisco, California, United States of America; 2 Department of Microbiology and Immunology, University of Rochester Medical Center, Rochester, New York, United States of America; Centro de Biología Molecular Severo Ochoa (CSIC-UAM), Spain

## Abstract

T-705 (Favipiravir) is a broad-spectrum antiviral molecule currently in late stage clinical development for the treatment of influenza virus infection. Although it is believed that T-705 potency is mediated by its ribofuranosyl triphosphate (T-705 RTP) metabolite that could be mutagenic, the exact molecular interaction with the polymerase of influenza A virus (IAVpol) has not been elucidated. Here, we developed a biochemical assay to measure the kinetics of nucleotide incorporation by IAVpol in the elongation mode. In this assay, T-705 RTP was recognized by IAVpol as an efficient substrate for incorporation to the RNA both as a guanosine and an adenosine analog. Compared to natural GTP and ATP, the discrimination of T-705 RTP was about 19- and 30-fold, respectively. Although the single incorporation of the ribonucleotide monophosphate form of T-705 did not efficiently block RNA synthesis, two consecutive incorporation events prevented further primer extension. In comparison, 3′-deoxy GTP caused immediate chain termination but was incorporated less efficiently by the enzyme, with a discrimination of 4,900-fold relative to natural GTP. Collectively, these results provide the first detailed biochemical characterization to evaluate the substrate efficiency and the inhibition potency of nucleotide analogs against influenza virus polymerase. The combination of ambiguous base-pairing with low discrimination of T-705 RTP provides a mechanistic basis for the in vitro mutagenic effect of T-705 towards influenza virus.

## Introduction

The influenza virus belongs to the *Orthomyxoviridae* family and is the causative agent of recurring seasonal and pandemic influenza outbreaks. There are currently only two classes of FDA-approved drugs for the treatment of influenza virus infection, the M2 ion channel blockers and the neuraminidase inhibitors. The M2 ion channel blockers amantadine and rimantadine are of limited use mainly due to rapid emergence of resistant viruses, to the point that currently circulating seasonal H1N1 and H3N2 influenza viruses harbor mutations in the M2 protein and are amantadine resistant [1]. Likewise, strains of influenza virus that are naturally resistant to the neuraminidase inhibitor oseltamivir have emerged in recent epidemics, such as the H1N1 virus circulating during the 2007–2008 and 2008–2009 seasons [2], [3]. Therefore, there is a clear need to develop new treatments to control seasonal and pandemic influenza, and also to identify new types of antiviral molecules that are effective against oseltamivir and adamantane-resistant viruses. The influenza A virus (IAV) contains eight segments of single-stranded RNA of negative polarity, encoding a total of at least ten different proteins. Among them, the IAV RNA-dependent RNA polymerase (RdRp) complex is formed by the association of PB1, PB2, and PA. Together, these three subunits are responsible for transcription and replication of the viral genome (vRNA). Once influenza A virus infects cells, the vRNA is transcribed into viral mRNA. This step requires the use of the cap structure of the host mRNA which is directly recognized by PB2 and cleaved by the endonuclease activity of PA [4], [5]. This enzymatic process, termed cap snatching, provides a capped primer for the PB1 subunit carrying the RdRp activity. The vRNA is also copied into its complementary positive strand RNA ((+) cRNA), and functions as a template intermediate to further amplify vRNA synthesis for virus progeny formation. The first 12–13 nucleotides at the 5′- and 3′- end of each RNA segment are extremely conserved [6], [7]. Partial base-pairing between the conserved 5′- and 3′-ends of the RNA forms a promoter region with a “panhandle” looped structure that regulates the initiation of transcription [8], [9]. According to this model, the 5′- terminus of the viral RNA plays an important role as a recognition element by influenza virus polymerase [10], [11]. In recent years, several groups have been able to produce and purify the recombinant IAV polymerase complex by co-expressing PB1, PB2, and PA genes [12], [13], [14], [15]. The RdRp activity of the resulting protein trimer was characterized by steady-state kinetics [15], and its enzymatic fidelity was characterized by measuring the level of mis-incorporation of natural nucleotides [12]. However, to our knowledge, there are no published studies that describe incorporation of nucleotide analogs which can be used as chain terminating inhibitors by recombinant IAV polymerase.

The antiviral 6-fluoro-3-hydroxy-2-pyrazinecarboxamide (T-705, favipiravir) is a nucleoside precursor entering phase III clinical trials in the United States for the treatment of influenza virus infections [16], [17]. *In vitro*, T-705 is efficiently converted to its ribofuranosyl 5′-triphosphate (T-705 RTP) form by cellular enzymes [18]. Recently, a new study showed that treatment of IAV-infected cells with T-705 resulted in a significant increase of lethal mutations within the viral genome [19]. In order to cause lethal mutagenesis, T-705 RTP would have to be recognized as a nucleotide substrate for IAVpol and be incorporated into the RNA in its monophosphate form (T-705 RMP) without causing immediate chain termination. Although T-705 RTP has been shown enzymatically to inhibit the RdRp activity of IAV polymerase [20], the exact mode of action and precise molecular interaction between the nucleotide and the viral enzyme has not yet been reported. A limitation to these studies is the lack of comprehensive enzymatic methods to monitor the kinetics of single nucleotide incorporation by IAV polymerase leading to either further primer extension or chain termination.

Here we report the detailed mechanism of inhibition of influenza A virus polymerase by T-705 RTP. We designed specific short RNA templates used in enzyme kinetic assays to probe the selectivity of recombinant IAVpol against different nucleotide analogs through single- and multiple-nucleotide incorporation experiments. We show that, (1) T-705 RTP is efficiently recognized by IAVpol both as a guanosine and as an adenosine analog, (2) single incorporation of T-705 RMP into RNA delays but does not block primer extension, and (3) two consecutive events of incorporation of T-705 RMP completely block subsequent RNA synthesis. Collectively, this study provides the first biochemical characterization of IAVpol interaction with T-705 RTP, further supporting the observation that T-705 could induce lethal mutagenesis during virus replication. In addition, our results offer new insights into the enzymatic fidelity of influenza virus polymerase, as they suggest that the overall error rate during RNA synthesis could be higher than previously measured.

## Materials and Methods

### Chemicals and Nucleic Acids

All NTPs were purchased as ultrapure grade from Affymetrix (Santa Clara, CA). Rabbit globin mRNA and heparin sodium salt were from Sigma-Aldrich. MgCl_2_, EDTA, NaCl solutions and Tris-Cl buffers were purchased from Life Technologies. Radiolabeled nucleoside triphosphates were purchased from Perkin Elmer. RNA oligonucleotides (sequences in table 1) were chemically synthesized by Dharmacon Inc. (Chicago, IL). 3′dGTP was purchased from Trilink (San Diego, CA), T-705 RTP and the nucleoprotein inhibitor were synthesized at Alios BioPharma.

**Table 1:** Sequences of RNA oligonucleotides used in this study.

Name	Sequence
t14-1 template	3′UCGAAAAAGCAAGG
t14-1 promoter	5′AGUAGAAACAGUUCC
t14-2 template	3′UCGAAAAAGUAAGG
t14-2 promoter	5′AGUAGAAACAAUUCC
t14-3 template	3′UCGAAAAAGUCCGG
t14-3 promoter	5′AGUAGAAACAAGGCC
50-nt template	5′UCGACAAUCAUCGGAUUGAAGCAUUGUCGCAAUCAGUACCUGCUUUCGCU
15-nt promoter	5′AGUAGAAACAAGGCC

### Expression and Purification of Recombinant IAV Polymerase Complex

Cloning, expression and purification of the influenza A virus PA/PB1/PB2 polymerase complex were performed as previously described [12]. In brief, the PA, PB1 and PB2 genes (from the H3N2 IAV strain (A/chicken/Nanchang/3-120/01)) were cloned into the baculovirus expression vector pVL1392 (Invitrogen) between the BglII and XbaI sites. The PA gene was tagged at the C-terminus with the tandem affinity purification (TAP) tag, which contains a thrombin cleavage site followed by 6xHis tag, tobacco etch virus (TEV) cleavage site and finally an IgG-binding domain. Each of the three pVL1392 plasmids was individually transfected into Sf9 insect cells for the production of recombinant viruses. The three recombinant viruses then co-infected Tni insect cells to obtain trimeric IAV polymerase complex. Cells were harvested 72 hours post infection and the lysates obtained were purified by the TAP purification technique [21].

### Polymerase Assay and Inhibition Testing using the Influenza Virus RNP Complex

Influenza virus (A/WSN/33 (H1N1)) was purchased from Virapur (San Diego, CA). 5×10^7^ IU/ml virus was incubated for 10 min in a reaction buffer containing 50 mM Tris-HCl, pH 8, 50 mM KOAc, 5 mM MgCl_2_, 1 mM DTT and 0.5% Triton X- 100. Viral genomic RNA transcription was started by mixing the lysed viruses with 12.5 ng/µl rabbit globin mRNA, 50 µM ATP, 50 µM UTP, 50 µM CTP, 2 µM GTP, and 0.17 µM [α-^33^P]GTP in the same reaction buffer at 37°C for 1 h. The reactions were quenched with a solution containing 90% formamide, 50 mM EDTA, 0.1% bromophenol blue and 0.1% xylene cyanol. The samples were denatured at 95°C for 5 min. The samples underwent electrophoresis on a 6% denaturing PAGE gel (Invitrogen) at 190 volts for 50 minutes.

### Inhibition and Competition Assays using Recombinant IAV Polymerase Complex

The activity of the purified polymerase complex was measured using the 5′-ApG replication assay as described previously [12]. For testing 3′dGTP and T-705 RTP inhibition effect, the reactions were performed at 37°C for 40 min in a reaction mixture containing reaction buffer (25 mM Tris-Cl, pH 7.5, 100 mM NaCl, 5 mM MgCl_2_, 0.5 mM EDTA, 2 mM DTT, 5% glycerol), 0.15 µM polymerase complex, 0.4 mM 5′-ApG primer, 1.5 µM 50-nt 3′vRNA template, 1.6 µM 15-nt 5′vRNA, 0.20 U/µl RNaseIn, 500 µM UTP, ATP, CTP, and 1 µM GTP mixed with 2.5 µCi [α-^33^P]GTP. The competition assays were performed under similar conditions, except that each competing nucleotide was tested at low (3 µM) or high (300 µM) concentration, while keeping the other NTPs at 300 µM. For the GTP and ATP competition assays, we replaced the isotope tracer to [α-^33^P]CTP. The reactions were stopped by the quench solution containing formamide with 50 mM EDTA. The samples were denatured at 95°C for 5 min. The samples were run on a 15% denaturing PAGE gel (Invitrogen) at 190 volts for 50 minutes.

### Single Nucleotide Incorporation Assay using Recombinant IAV Polymerase Complex

The recombinant polymerase complex catalyzed the primer extension reaction using 5′-pApG as primer, a 14-nt synthetic RNA oligonucleotide as template and a 15-nt RNA as promoter. The 14-nt RNA template and 15-nt RNA promoter form a panhandle structure that was required for RNA replication activity of the influenza polymerase complex [10], [22]. To study if T-705 RTP can be incorporated opposite U or C on the template, nucleotide incorporation assays using specific templates were performed. The sequences of RNA oligos used in this experiment are listed in Table 1. A typical reaction was performed at 37°C in a reaction mixture containing reaction buffer (40 mM Tris-HCl, pH7, 20 mM NaCl, 5 mM MgCl_2_, and 2 mM DTT), 0.15 µM polymerase complex, 1 µM 15-nt RNA promoter, 2.5 µM 14-nt RNA template, 0.4 mM 5′-pApG primer, 0.2 U/µl RNaseIn, 25 µM UTP, 25 µM CTP, 0.033 µM [α-^33^P]CTP, and other NTPs at various concentrations as indicated in figure legends. The replication reactions were stopped after 35 min by mixing with 2x volume of the formamide quench solution. The quenched reactions were denatured at 95°C for 3 min and then were loaded onto a 22.5% denaturing polyacrylamide gel with 7 M urea (National Diagnostics, Atlanta, GA). The electrophoresis was performed at 80 watts using a Sequi-Gen GT system from Bio-Rad (Hercules, CA).

### Product Analysis

Denaturing PAGE gels were dried at 80°C for one hour with a Model-583 gel drier (BioRad, Hercules, CA). Dried gels were exposed to storage phosphor screens and visualized by a Typhoon scanner (GE healthcare). The fraction of RNA products were calculated based on the intensities of bands on the gel that were quantified using the ImageQuant software (GE Healthcare).

### Data Analysis

IC_50_- The compound concentration at which the enzyme-catalyzed rate was reduced by 50% (IC_50_) was calculated by fitting the data to the equation
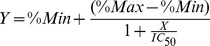



Where Y corresponds to the percent relative enzyme activity, % Min is the residual relative activity at saturating compound concentration, % Max is the relative maximum enzyme activity, and X corresponds to the compound concentration. The IC_50_ value was derived from the mean of a minimum of two independent experiments.

*K*_app_ - The phosphor images of the reactions were analyzed by ImageQuant software. For each lane, the intensity for each band starting from 9-mer up to 14-mer was quantified. The percentage of the bands intensity (10-mer to 14-mer) in the total intensity (9-mer to 14-mer) represented the percentage of the products converted from 9-mer. The data of concentration dependent 9-mer conversion to product were fit to a hyperbolic equation to obtain the *K*_app_ concentration.
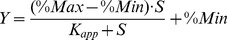



Where *Y* is the percentage of all observed products, *%Min* is the percent product from mis-incorporation when the nucleotide in the study was not added, *%Max* is the maximal product percentage, *S* is the concentration of NTP in the study, and *K*_app_ is the concentration of NTP when half of the 9-mer was converted to products. All *K*_app_ values were derived from the mean of a minimum of two independent experiments.

Discrimination defines the relative incorporation efficiency of a nucleotide analog or an incorrect nucleotide to the incorporation efficiency of the natural correct nucleotide. According to our equation derivation (see Appendix S1 in File S1), the discrimination of IAVpol between natural correct nucleotide (*correct*) and an analog or incorrect nucleotide (*NTP*) can be calculated as
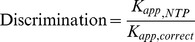



Where *K*_app,NTP_ is the *K*_app_ for an NTP under study and *K*_app,correct_ is the *K*_app_ of the correct natural nucleotide for incorporation at position 10. Similarly, the fidelity or error rate (one error per incorporation site) for IAVpol can be calculated as
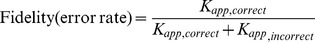



## Results

### Inhibition of the Polymerase Activity of the Influenza Virus RNP Complex

**Figure 1:**
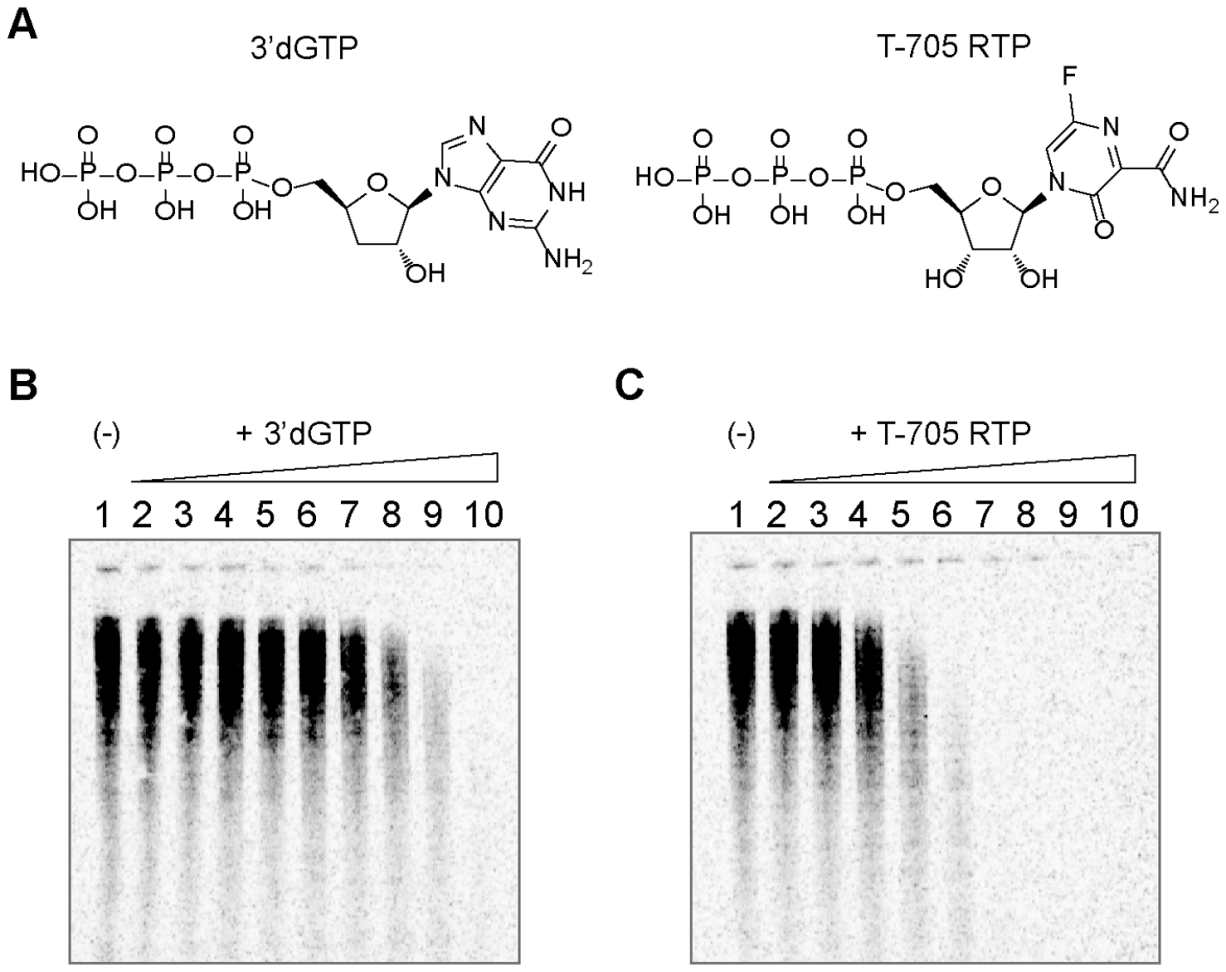
Inhibition of the influenza virus RNP complex by 3′dGTP and T-705 RTP. (A) Chemical structures of the obligate chain terminator 3′dGTP and the base-modified T-705 ribofuranosyl 5′-triphosphate (T-705 RTP). (B) Polyacrylamide gel electrophoresis (6%) showing the decrease in radiolabeled viral RNA product from the enzymatic reaction in the presence of increasing concentrations of 3′dGTP. Concentrations of inhibitor are as follows: lane 1 (0), lane 2 (0.023), lane 3 (0.069), lane 4 (0.21), lane 5 (0.62), lane 6 (1.9), lane 7 (5.6), lane 8 (16.7), lane 9 (50), and lane 10 (150 µM). (C) same as (B), with the same concentration range of T-705 RTP as inhibitor.

We initially evaluated two nucleotide analogs against the RdRp activity of viral ribonucleoprotein (RNP) complex prepared from purified influenza virus particles. We used 3′dGTP as a positive control for polymerase inhibition because it is an obligate chain terminator [23] (Fig. 1A). Under our enzyme assay conditions, increasing concentrations of 3′dGTP resulted in progressive inhibition of radio-labeled RNA synthesis (Fig. 1B). A nucleozin analog used as a nucleoprotein inhibitor, and therefore acting by a different mechanism, also inhibited RNA synthesis (Fig. S1 in File S1) [24], [25]. Similarly, T-705 RTP inhibited the RdRp activity of influenza virus vRNPs, but the low resolution of the RNA separation on the gel did not reveal any clear chain termination products (Fig. 1C). Notably, T-705 RTP was significantly more potent than 3′dGTP when tested under the same concentration range. The possibility that T-705 RTP could act as a chain terminator of influenza virus RdRp activity was further explored in the next experiments.

### T-705 RTP Inhibits Recombinant IAVpol by Competing Against GTP and ATP

**Figure 2:**
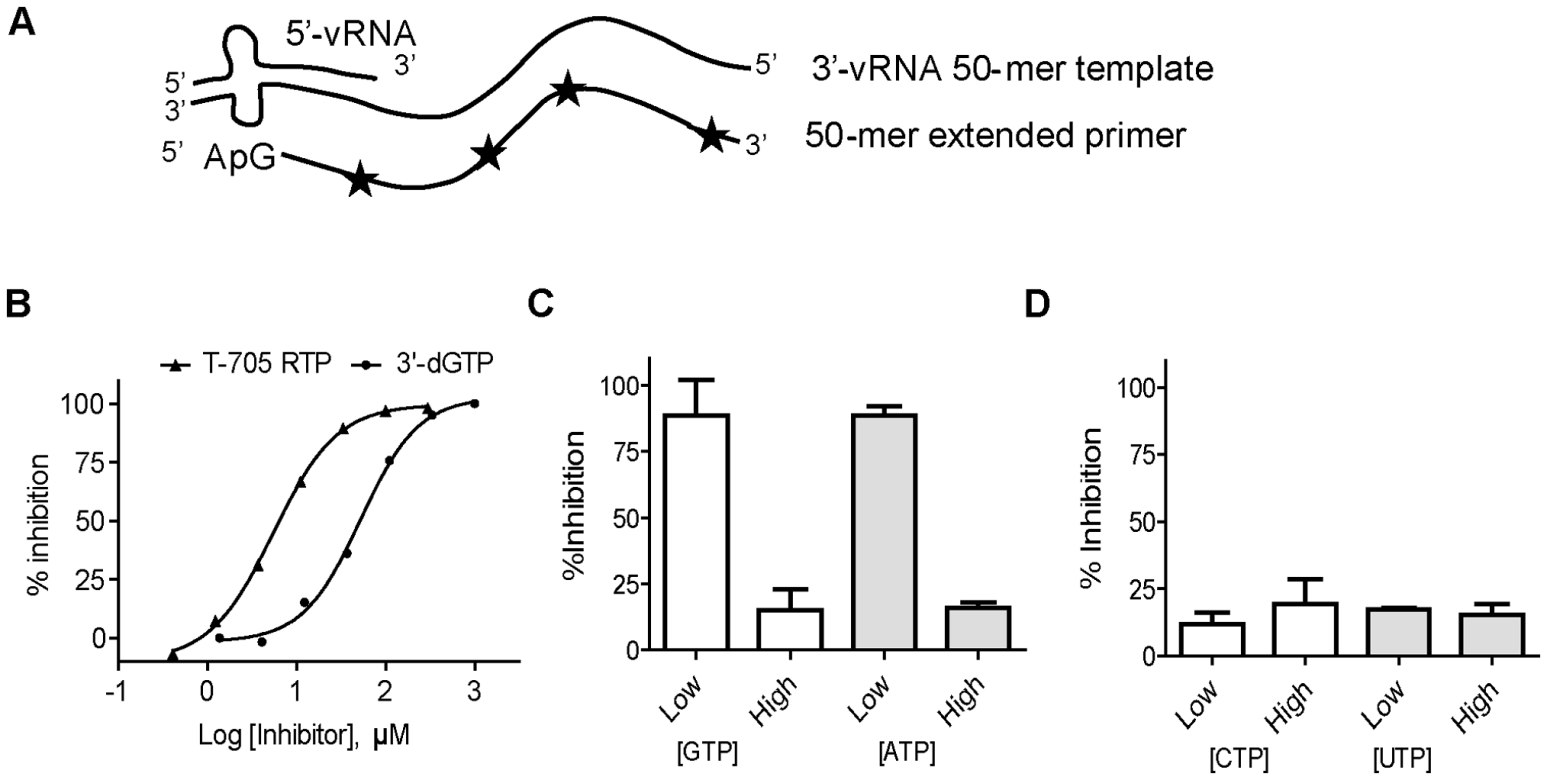
Inhibition of recombinant IAVpol using a 50-mer RNA template. (A) Principle of the reaction. Recombinant IAVpol (PA/PB1/PB2) was incubated in the presence of a 50-mer RNA template sequence derived from the 3′-end of the PA gene of the NanChang strain [12]. The 15-nt 5′vRNA oligo that is partially complementary to the 3′vRNA is needed as promoter for the enzyme. The 5′-pApG dinucleotide primer is extended and allows for multiple incorporation events of α-^33^P-GMP used as tracer (star). (B) Representative curves of inhibition potency of 3′dGTP and T-705 RTP against IAVpol RNA synthesis activity. IC_50_s were determined by adding increasing concentrations of each inhibitor, and quantitative analysis of the amount of remaining full length RNA product is expressed as % inhibition (see Materials and Methods). Each experiment was conducted at least twice to calculate the average value and standard deviation. (C) The inhibition percentage was measured in the presence of a saturating concentration of T-705 RTP (100 µM), and either low (3 µM) or high (300 µM) concentration of one of the two purines GTP or ATP. (D) The same experiment as (C) instead with either low (3 µM) or high (300 µM) of pyrimidine UTP or CTP.

Next, we used the recombinant IAVpol trimer purified from insect cells following previously described methods [12]. The enzymatic reaction was initiated after recognition of the promoter loop structure by the enzyme, and the ApG dinucleotide primer was extended to a 50-mer RNA product (Fig. 2A). We modified the assay by reducing the concentration of natural GTP down to 1 µM in order to favor competition with GTP analogs. Under these conditions, both 3′dGTP and T-705 RTP inhibited the RdRp activity of IAVpol with an IC_50_ of 43±12 and 2.9±0.14 µM, respectively (Fig. 2B). As expected, increasing the concentration of natural GTP to 300 µM almost completely prevented the inhibition of polymerase activity with 3′dGTP (data not shown) and T-705 RTP (Fig. 2C). Increasing the concentration of natural ATP to 300 µM also produced the same loss of inhibition effect on T-705 RTP. In comparison, adding CTP and UTP at varying concentrations did not change the inhibition potency of T-705 RTP (Fig. 2D). These results suggest that T-705 RTP is recognized by IAVpol as an ambiguous nucleotide acting both as a guanosine and an adenosine analog.

### Single GMP and T-705 RMP Incorporation Opposite Cytidine on Template

**Figure 3:**
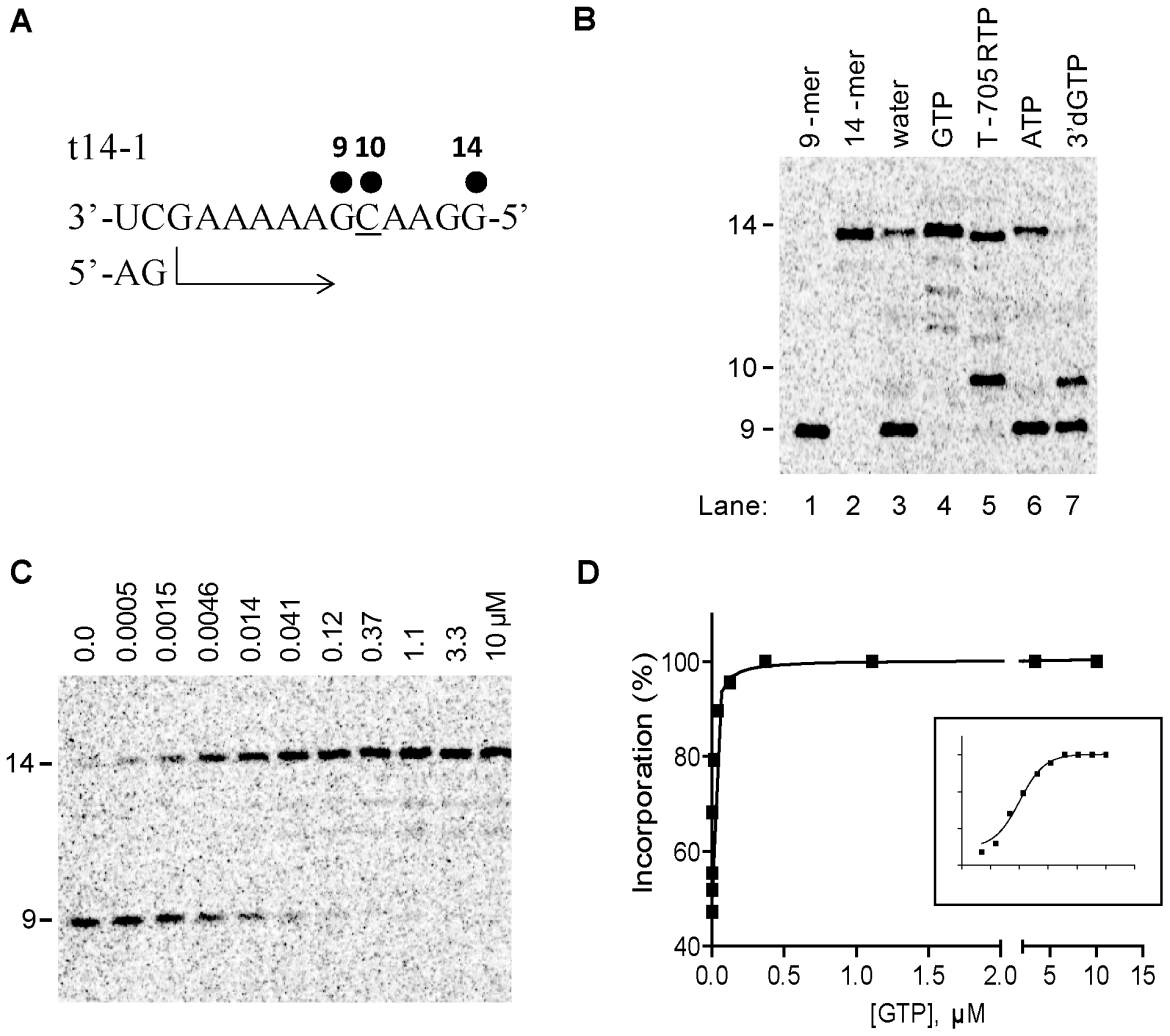
GMP and T-705 RMP incorporation opposite C on template. (A) A 14-mer RNA template sequence (t14-1) was designed to favor the extension of the 5′-pApG dinucleotide primer (AG) to a 9-mer in the presence of CTP and UTP, or a 14-mer full-length product (FL) in the presence of the additional GTP that can be specifically incorporated at position +10. (B) Example of a polyacrylamide gel electrophoresis result showing the products of primer extension. In addition to 25 µM CTP and UTP, 100 µM of GTP (lane 4), T-705 RTP (lane 5), ATP (lane 6), or 3′dGTP (lane 7) were added to the enzymatic reaction. The 9-mer and 14-mer product sequences were chemically synthesized, radiolabeled, and used as molecular size markers during the gel migration (lane 1 and 2). (C) Natural GTP was added to the primer extension reaction at increasing concentrations up to 10 µM in the presence of 25 µM CTP and UTP. (D) Quantitative analysis of GMP incorporation, based on the extension of the 9-mer RNA product obtained in Fig. 3C. The percentage of the extended products from 9-mer was plotted against GTP concentration and the data was fitted to a hyperbolic equation (see Materials and Methods) to derive the *K*_app_ for GTP incorporation. The inset shows the same plot on semi-log scale. Each experiment was conducted at least twice to calculate the average value and standard deviation (see Table 2).

We further modified the enzymatic assay by shortening the size of the template strand to a 14-mer RNA (t14-1). This new template sequence was designed to maintain partial base-pairing with the modified 5′vRNA complementary strand (t14-1 promoter), while allowing for single incorporation of GMP or GMP analogs at position +10 (Fig. 3A). When we supplied the enzymatic reaction with CTP and UTP, RNA synthesis was mainly stalled at position +9 (Fig. 3B, lane 3). We noticed a small amount of full length extension to position +14 in the absence of GTP, likely due to residual mis-incorporation events opposite C on the template. This level of mis-incorporation was slightly increased in the presence of 100 µM of ATP (lane 6). When GTP was provided to the reaction at a fixed concentration of 100 µM in addition to CTP and UTP, the 9-mer product disappeared on the gel and was converted to the 14-mer full length extension product (lane 4). Adding T-705 RTP instead of natural GTP also resulted in the conversion of the 9-mer to 14-mer, with an additional 10-mer product (lane 5). As expected, the obligate chain terminator 3′dGTP produced only a 10-mer chain termination product (lane 7). Moreover, it prevented the formation of the full-length mis-incorporation product observed in lane 3.

We then determined the relative efficiency of substrate incorporation of natural GTP and other NTP analogs by measuring the apparent nucleotide concentration at which half of the maximum product formation was reached during the elongation phase, or *K*app (Fig. 3C and D). Although the enzyme-RNA elongation complex *per se* could not be kinetically separated from the slow and abortive initiation step, we were able to derive the simple relationship between *K*app and the individual kinetic parameters of nucleotide incorporation *kpol* and *Kd* (see equations in Appendix S1 in File S1). We measured that the *K*app value for GTP was 0.0078 ± 0.003 µM (Table 2). We repeated the same experiment with the other NTPs in order to compare their relative efficiency of incorporation, expressed as discrimination (*K*app, GTP/*K*app, NTP). We found that T-705 RTP was discriminated only by 19-fold, compared to 330,000- and 4,900-fold for ATP (A:C mismatch) and 3′dGTP, respectively (Table 2). These results show a good correlation between the high enzyme inhibition potency of T-705 RTP as measured in a competition assay (IC50) and its high substrate efficiency (or low discrimination), compared to 3′dGTP.

**Table 2:** Enzymatic efficiency of single NMP incorporation opposite cytidine (N:C).

RNA template: 3′-UCGAAAAAGCAAGG-5 (t14-1)				
	GTP	T-705 RTP	ATP	3′dGTP
*K_app_* (µM)	0.0078±0.003	0.15±0.04	2,560±720	38±34
Discrimination*	1	19	330,000	4,900

* calculated as *K*_app,NTP_/*K*_app,GTP._

### Single AMP and T-705 RMP Incorporation Opposite Uridine on Template

**Figure 4:**
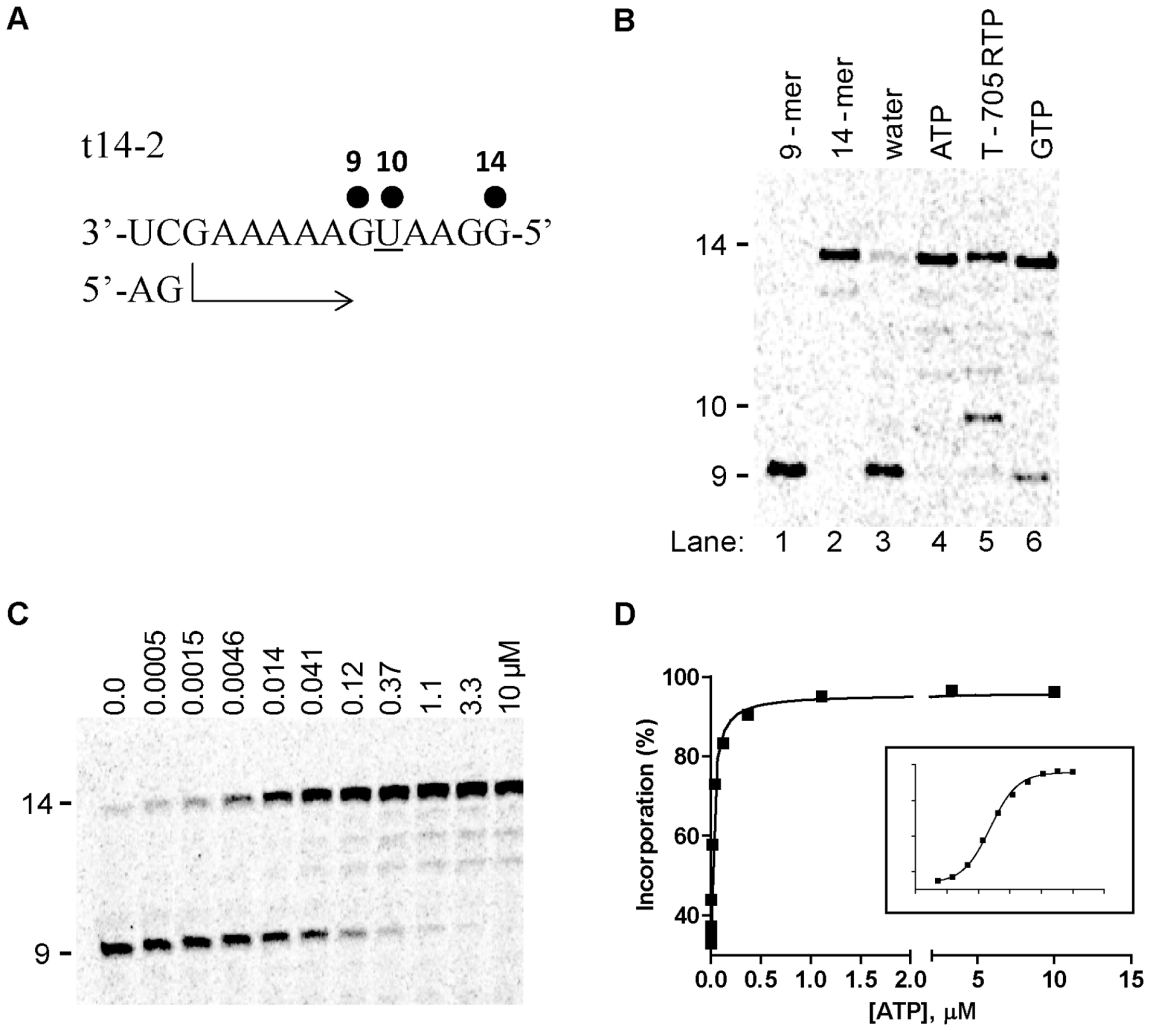
AMP and T-705 RMP incorporation opposite U on the template. (A) A 14-mer RNA template sequence (t14-2) was designed to favor the extension of the 5′-pApG dinucleotide primer (AG) to a 9-mer in the presence of CTP and UTP, or a 14-mer full-length product (FL) in the presence of additional ATP that can be specifically incorporated at position +10. (B) Polyacrylamide gel electrophoresis result showing the product of primer extension in the presence of 100 µM of either ATP (lane 4), T-705 RTP (lane 5), or GTP (lane 6). The 9-mer and 14-mer product sequences were chemically synthesized, radiolabeled, and used as molecular size markers (lane 1 and 2). (C) Natural ATP was added to the reaction at increasing concentrations up to 10 µM. (D) Quantitative analysis of AMP incorporation, based on the extension of the 9-mer RNA product obtained in (C). The percentage of the extended products from 9-mer was plotted against ATP concentration, and the data was fit to a hyperbolic equation (see Materials and Methods) to derive the *K*_app_ for ATP incorporation. The inset shows the same plot on semi-log scale. Each experiment was conducted at least twice to calculate the average value and standard deviation (see Table 3).

We followed the same methodology as described above, but we modified the template sequence to accommodate a single adenosine opposite U at position +10 (t14-2), instead of guanosine opposite C (Fig. 4A). We supplied the enzymatic reaction with the required NTP substrates except ATP, and RNA synthesis was stalled at position +9. A small observable amount of full length extension was found, and this was most likely due to residual mis-incorporation products (Fig. 4B, lane 3). When ATP was provided to the reaction at a fixed concentration of 100 µM, the 9-mer product disappeared and the RNA was converted to 14-mer full length product (lane 4). Adding T-705 RTP instead of natural ATP also resulted in the conversion of the 9-mer to 10- and 14-mer products (lane 5), in agreement with our initial ATP competition results. Interestingly, GTP opposite U (G:U mismatch) also gave a robust 14-mer RNA product, indicative of efficient mis-incorporation of this nucleotide at the +10 position (lane 6). We also determined the substrate efficiency of natural ATP and the other two nucleotides by measuring the *K*_app_ at the 9-mer position (Fig. 4C and D, values summarized in Table 3). Using the same methodology as described for GTP opposite C, we found that the *K*_app_ value for ATP was 0.022±0.003 µM^−1^ (Table 3). We calculated that GTP opposite U (G:U mismatch) is only discriminated by 420-fold, which reflects its relatively high substrate efficiency of mis-incoporation in the absence of the correct nucleotide. In comparison, T-705 RTP opposite U was discriminated only by 30-fold, a number that is similar to what we previously measured for T-705 RTP opposite C.

**Table 3:** Enzymatic efficiency of single NMP incorporation opposite uridine (N:U).

RNA template: 3′-UCGAAAAAGUAAGG-5 (t14-2)				
	ATP	T-705 RTP	GTP	
*K_app_* (µM)	0.022±0.003	0.66±0.39	9.2±0.54	
Discrimination*	1	30	420	

* calculated as *K*_app,NTP_/*K*_app,ATP._

### Effect of Multiple Incorporations of T-705 RMP on Primer Extension

**Figure 5:**
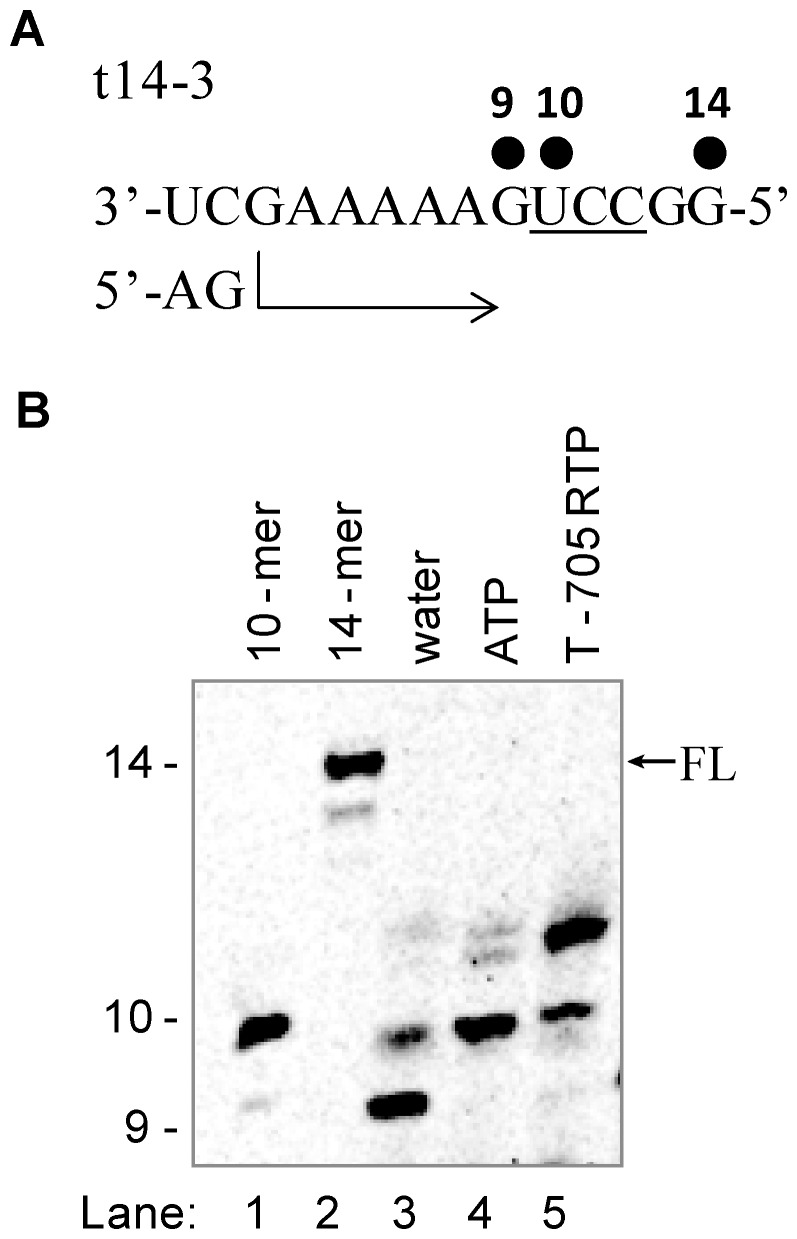
Effect of multiple incorporations of T-705 RMP on RNA synthesis. (A) A 14-mer RNA template sequence (t14-3) was designed to favor the extension of the 5′-pApG dinucleotide primer (AG) to a 9-mer in the presence of CTP and UTP, or a 10-mer product in the presence of additional ATP that can be specifically incorporated at position +10. (B) Polyacrylamide gel electrophoresis result showing the product of primer extension in the presence of 100 µM of ATP (lane 4) or T-705 RTP (lane 5). The 10-mer and 14-mer, representing the full length product (FL), RNA sequences were chemically synthesized, radiolabeled, and used as molecular size markers (lane 1 and 2).

We designed a third 14-mer RNA template sequence (t14-3) containing a series of 3 pyrimidines (UCC) at positions +10, +11, and +12 (Fig. 5A). When just CTP and UTP were added in the primer extension reaction, a majority of the RNA product remained at position +9 with some minor amounts of mis-incorporation (lane 3). Once CTP, UTP and ATP were added to the reaction, most of the 9-mer was converted to 10-mer (lane 4). The observed *K*_app_ of AMP with this new sequence was 0.038 µM, a value that is similar to the result obtained with the t14-2 RNA template (Fig. S2 in File S1). Adding T-705 RTP instead of ATP did not result in partial inhibition of the full-length 14-mer product as seen in Fig. 3B and 4B. Instead, we observed a distinct pattern of +10 and +11 additions, indicating that only two consecutive molecules of T-705 RMP could be incorporated into the RNA, and these products resulted in complete inhibition of further nucleotide incorporation (lane 5). These results suggest that T-705 RMP at the 3′end of the nascent RNA acted as a non-immediate or “leaky” chain terminator, and this inhibition effect was amplified by two consecutive events of T-705 RMP incorporation.

## Discussion

T-705 is currently the most advanced small molecule clinical candidate for the treatment of influenza A and B virus [26]. It is widely believed that T-705 exerts its antiviral activity through its nucleoside triphosphate form (T-705 RTP) by directly inhibiting the RdRp activity of IAV polymerase [20], but the exact mode of action and precise molecular interaction between the nucleotide and the viral polymerase has not yet been reported.

The aim of our study was to develop novel enzymatic methods to specifically measure the interaction between T-705 RTP and the influenza virus polymerase. A challenge we faced in the design of short RNA templates suitable for single nucleotide incorporation assays was the relatively stringent sequence requirements from the promoter elements needed to maintain the panhandle structure of the 5′vRNA with the template strand [10], [22]. Any disruption of these secondary hairpin loop structures, or other base-pairing interactions between the 5′vRNA and the 3′-end of the template, usually results in a complete loss of polymerase activity. Following the relationship between changes in RNA sequence and the polymerase activity observed by Fodor *et al*. [10], we were able to design a template and promoter pair that can be used to study the specific single nucleotide incorporation of natural GTP and GTP analogs (Fig. 3). We originally optimized the conditions of the enzymatic reaction with 3′dGTP, an obligate chain terminator due to its lack of 3′OH group. However, the use of 3′dGTP as an inhibitor of IAVpol was limited due to weak potency across the different sources of enzymes and RNA templates (Fig. 1 and 2). This lack of potency was explained by the low substrate efficiency of - or high discrimination against - the incorporation of 3′dGMP, as measured in our single nucleotide incorporation assay (Fig. 3 and Table 2). In comparison, T-705 RTP was a more efficient substrate for IAVpol, which correlates well with the potent inhibition we observed against virus-derived RNP complex and the recombinant IAV polymerase PA/PB1/PB2 complex. Additional kinetic studies of single nucleotide incorporation further validated the relationship between substrate efficiency and inhibition potency. Potent inhibitors of hepatitis C virus polymerase, such as 3′dATP and the 2′-methyl ribonucleotides, were not significantly incorporated by IAVpol and did not significantly reduce its activity in our single nucleotide incorporation assay (data not shown). This assay was also used to measure the efficiency of mis-incorporation for ATP opposite cytidine (A:C mismatch) and GTP opposite uridine (G:U mismatch) (Table 2 and 3). The enzymatic error rate of RNA synthesis by IAV polymerase at a given site can be calculated using measured *K*_app_ (see Materials and Methods). In our case this translated into an error rate of 3.1×10^−6^ for A:C, and 2.4×10^−3^ for G:U. By comparison, the RNA polymerase of hepatitis C virus has an error rate of 2.3×10^−6^ for A:C, and 8.7×10^−3^ for G:U [27]. Other enzyme kinetic studies performed with viral RNA polymerases reported similar error rates [28], [29]. Therefore, we conclude that the fidelity of IAV polymerase is likely within the same range as for other viral RdRps, and perhaps lower than previously estimated [12].

**Figure 6:**
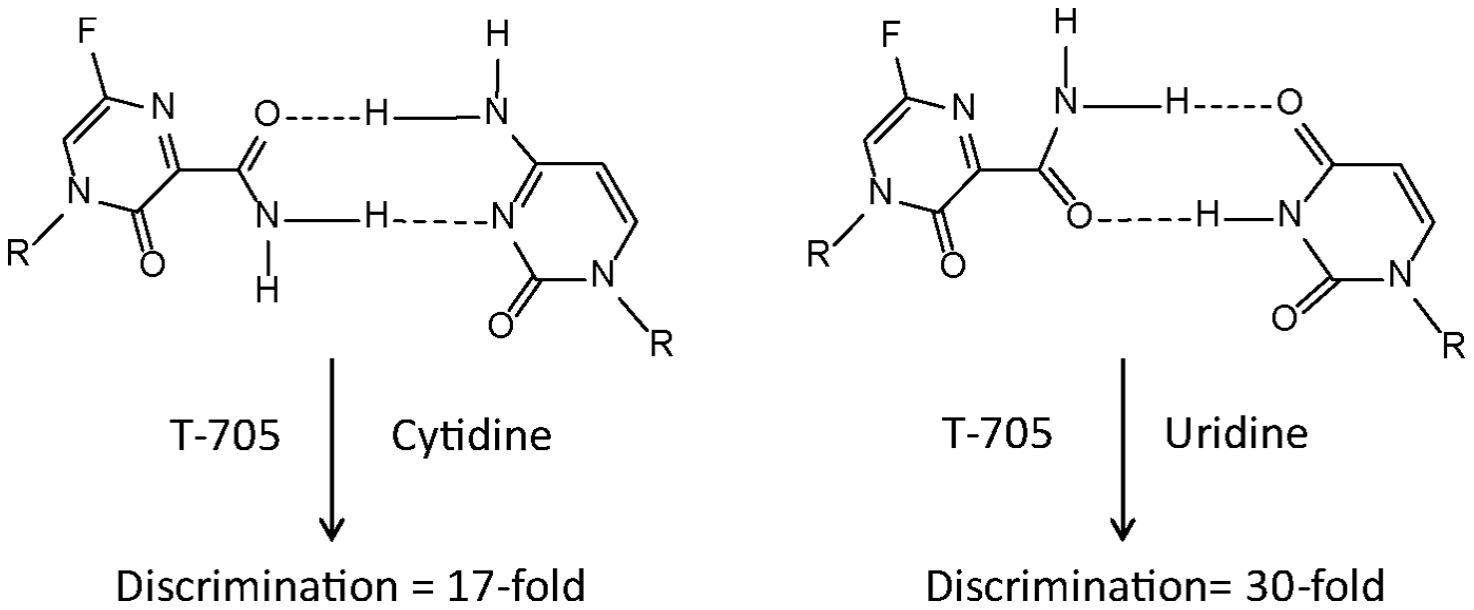
Schematic of ambiguous base pairing of T-705 opposite cytidine and uridine on RNA template. Enzyme kinetics of single nucleotide incorporation combined with competitive inhibition experiments demonstrated that T-705 RTP is able to base pair with a cytidine and a uridine prior to being incorporated by influenza virus polymerase with low discrimination against natural GTP or ATP.

Our present study also aimed to address the mechanism of action of T-705 by evaluating its nucleobase selectivity for IAV polymerase. Our results conclusively determined that T-705 RTP is an ambiguous base-pairing nucleotide which can be recognized by the viral polymerase as a guanosine and as an adenosine analog (summarized in Fig. 6). These results agree with our initial findings showing that T-705 RTP inhibition effect is competitive with ATP and GTP, but not with UTP or CTP (Fig. 2). Our results are also supported by the recent study showing that T-705 induces lethal mutagenesis in influenza virus *in vitro* [19]. The reported genome sequence analysis of virus treated with T-705 demonstrated enrichment of G-to-A and C-to-U transversion mutations, which is expected from ambiguous base-pairing against C and U. Collectively these findings are reminiscent of what has been previously reported for ribavirin triphosphate when tested against hepatitis C virus and poliovirus polymerase [30], [31], [32], [33]. Similar enzymatic results were used to explain the mutagenic effect of ribavirin *in vitro* when tested against poliovirus, arenavirus, and West Nile virus [33], [34], [35]. T-705 ribosyl and ribavirin each contain a carboxamide-modified nucleobase, and the possibility that they might share similar mechanistic antiviral effects, such as lethal mutagenesis, should be further explored. The low levels of discrimination of T-705 RTP by IAVpol support the hypothesis that high incorporation efficiency is important in order to induce a mutagenic effect during virus replication. In contrast, ribavirin-TP was incorporated by poliovirus polymerase with low efficiency, at a level similar to mis-incorporation [30], [31]. This could explain why T-705 appears more potent than ribavirin when tested side by side [20], [36]. It has been proposed that the low substrate efficiency of ribavirin-TP is compensated by the concomitant inhibition of IMPDH by ribavirin-MP resulting in a lower GTP pool, thereby increasing the frequency of incorporation of the nucleotide analog by viral polymerases [31]. The proposed mechanism(s) of action of ribavirin is a highly debated and controversial issue, and the fact that ribavirin has a broad antiviral spectrum could explain how different observed effects depend on the virus studied (for review: [37]). Likewise, T-705 has been shown to inhibit a number of unrelated RNA viruses, including Orthomyxoviruses, Noroviruses, Bunyaviruses, Arenaviruses, and Flaviviruses [38], [39], [40], [41], [42], [43], [44]. Similar to ribavirin, T-705 could alter the nucleotide pools resulting in an increase in mis-incorporation of incorrect nucleotides that could explain the observed mutagenic effect. In addition to the observation that T-705 might induce lethal mutagenesis in influenza virus due to its ambiguous base-pairing properties during RNA polymerization, we report that two consecutive incorporation events of T-705 RMP to the nascent RNA results in chain termination (Fig. 5). Our hypothesis is that while low levels of incorporation of T-705 RMP could result in full-length extension of viral RNA, leading to lower infectivity and lethal mutagenesis, too many consecutive events of incorporations would result in an antiviral effect by chain termination. However, the probability to incorporate successively two modified nucleotides during viral replication is relatively low. Therefore, it is still not clear if chain termination with T-705 RMP resulting in abortive RNA products is really a dominant effect. So far all attempts to apply selection pressure to influenza virus with repeated passages in the presence of T-705 have failed to select escape mutants [18], [19], [45]. In this context, more work needs to be done in order to fully understand the implications of our biochemical results and their relevance to virus inhibition and the clinical efficacy of T-705. In particular, the incorporation of T-705 ribunucleoside monophosphate into IAV genome within infected cells remains to be demonstrated. Like ribavirin, T-705 will also have to be carefully monitored for its potential to be incorporated into host cellular DNA and RNA, as well as its role, if any, as an immunomodulator [37], [46].

## Supporting Information

File S1 **Contains: Figure S1.** **Inhibition of influenza virus RNPs by a nucleoprotein binder.** Polyacrylamide gel electrophoresis (6%) result showing the decrease in radiolabeled viral RNA product from the enzymatic reaction in the presence of increasing concentrations of a nucleozin analog used as nucleoprotein inhibitor. Concentrations of inhibitor are as follows: lane 1 (0), lane 2 (0.015), lane 3 (0.046), lane 4 (0.14), lane 5 (0.41), lane 6 (1.2), lane 7 (3.7), lane 8 (11.1), lane 9 (33.3), and lane 10 (100 µM). **Figure S2. Determination of ATP substrate efficiency using t14-3 RNA Template.** (A) Natural ATP was added to the reaction at increasing concentrations up to 10 µM and the quantitative analysis of AMP incorporation based on the extension of the 9-mer RNA product to the +10 position. (B) The percentage of the extended products from 9-mer was plotted against ATP concentration and the data was fitted to a hyperbolic equation (see Materials and Methods) with a derived *K*_app_ = 0.038 µM for ATP incorporation. The inset shows the same plot on a semi-log scale. **Appendix S1. Equation derivation.**
(PDF)
