# Supplementary material for: The Ambiguous Base-Pairing and High Substrate Efficiency of T-705 (Favipiravir) Ribofuranosyl 5′-Triphosphate towards Influenza A Virus Polymerase
Source: PLoS One. 2013 Jul 10;8(7):e68347. doi: 10.1371/journal.pone.0068347 (PMC3707847; doi:10.1371/journal.pone.0068347)
Supplement: File S1 — Contains: Figure S1. Inhibition of influenza virus RNPs by a nucleoprotein binder. Polyacrylamide gel electrophoresis (6%) result showing the decrease in radiolabeled viral RNA product from the enzymatic reaction in the presence of increasing concentrations of a nucleozin analog used as nucleoprotein inhibitor. Concentrations of inhibitor are as follows: lane 1 (0), lane 2 (0.015), lane 3 (0.046), lane 4 (0.14), lane 5 (0.41), lane 6 (1.2), lane 7 (3.7), lane 8 (11.1), lane 9 (33.3), and lane 10 (100 µM). Figure S2. Determination of ATP substrate efficiency using t14-3 RNA Template. (A) Natural ATP was added to the reaction at increasing concentrations up to 10 µM and the quantitative analysis of AMP incorporation based on the extension of the 9-mer RNA product to the +10 position. (B) The percentage of the extended products from 9-mer was plotted against ATP concentration and the data was fitted to a hyperbolic equation (see Materials and Methods) with a derived K app = 0.038 µM for ATP incorporation. The inset shows the same plot on a semi-log scale. Appendix S1. Equation derivation. (PDF) [file pone.0068347.s001.pdf]

File S1

The Ambiguous Base-Pairing and High Substrate Efficiency of T-705 (Favipiravir) Ribofuranosyl 5'-triphosphate towards Influenza A Virus Polymerase

Zhinan Jin[1*], Lucas K. Smith[1], Vivek K. Rajwanshi[1], Baek Kim[2], and Jerome Deval[1*]

Figure S1

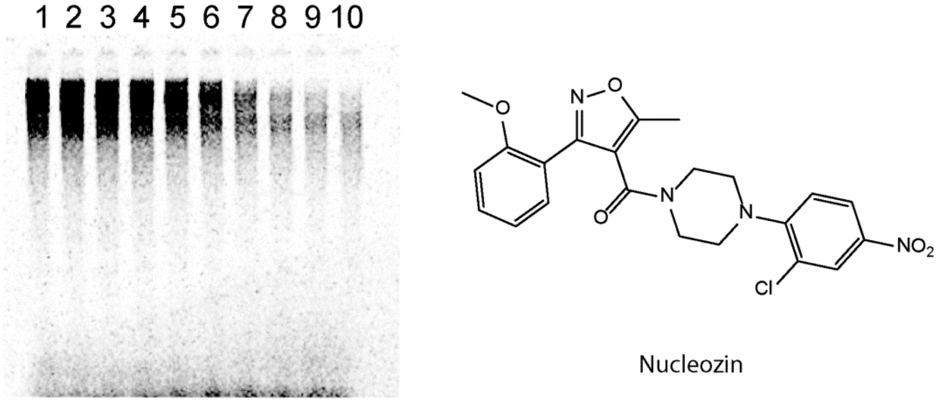

Figure S1. Inhibition of influenza virus RNPs by a nucleoprotein binder. Polyacrylamide gel electrophoresis (6%) result showing the decrease in radiolabeled viral RNA product from the enzymatic reaction in the presence of increasing concentrations of a nucleozin analog used as nucleoprotein inhibitor. Concentrations of inhibitor are as follows: lane 1 (0), lane 2 (0.015), lane 3 (0.046), lane 4 (0.14), lane 5 (0.41), lane 6 (1.2), lane 7 (3.7), lane 8 (11.1), lane 9 (33.3), and lane 10 (100 µM).

Figure S2

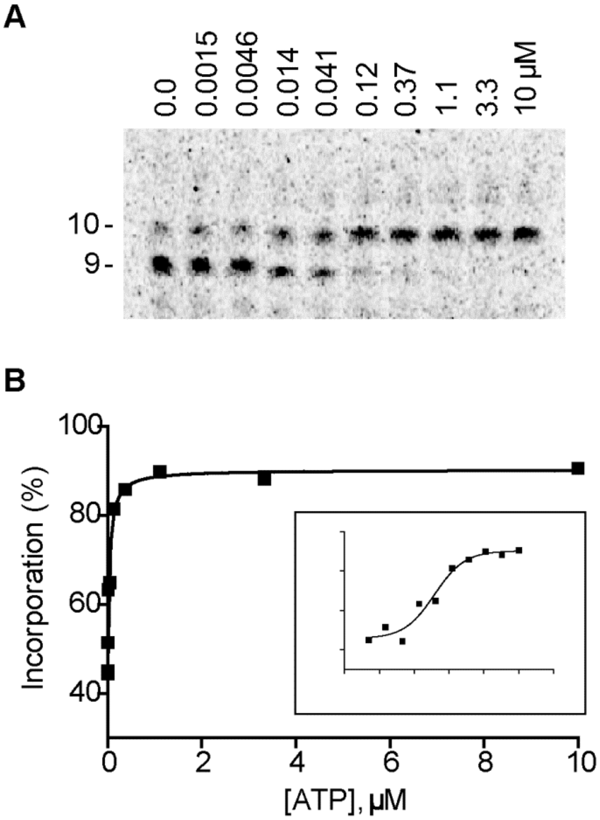

Figure S2. Determination of ATP substrate efficiency using t14-3 RNA Template. (A) Natural ATP was added to the reaction at increasing concentrations up to 10 μM and the quantitative analysis of AMP incorporation based on the extension of the 9-mer RNA product to the +10 position. (B) The percentage of the extended products from 9-mer was plotted against ATP concentration and the data was fitted to a hyperbolic equation (see Material and Methods) with a derived K_{app}= 0.038 μM for ATP incorporation. The inset shows the same plot on a semi-log scale.

Appendix S1. Equation derivation

For RNA synthesis catalyzed by the influenza RNA polymerase as shown in Figure S2, when pApG, CTP and UTP were added in the reaction, the enzyme binds to the dinucleotide primer first, and then adds nucleotide one by one to synthesize RNA products. The model of RNA synthesis from initiation to elongation to make the 9-mer product can be simplified to the model shown below. Nucleotide binding steps are not shown since these steps usually are rather fast (diffusion limited).

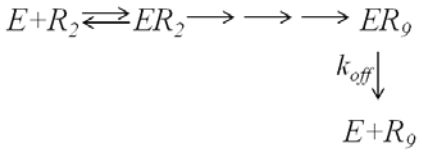

When the third nucleotide, ATP, is added into the reaction at various concentrations, 10-mer product is formed (see the model below).

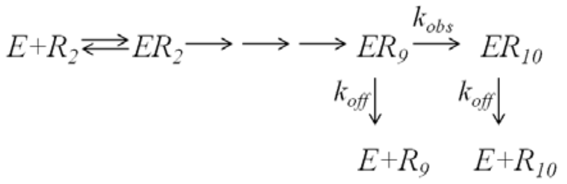

In this case, each ER_9 either coverts to ER_{10} or dissociates to release E and R_9. The conversion from ER_9 to ER_{10} follows this two step nucleotide incorporation mechanism during elongation mode,

$$ER_9 + NTP \xrightleftharpoons{K_d} ER_9 \cdot NTP \xrightleftharpoons{k_{pol}} ER_{10} + PP_i$$

Where k_{pol} is the maximal rate of nucleotide incorporation and K_d is the ground state binding equilibrium constant of the incoming nucleotide. The constant, k_{pol}/K_d, defines the specificity of nucleotide incorporation. From this mechanism, the observed rate of nucleotide incorporation (k_{obs}) at a certain concentration of the nucleotide substrate, S, follows this equation,

$$k_{obs} = \frac{k_{pol} S}{K_d + S}$$

When S increases, k_{obs} increases. At certain concentration S_0, k_{obs} reaches k_{off}, and the total amount of 9-mer (ER_9+R_9) formed during the reaction time equals to the amount of total 10-mer (ER_{10}+R_{10}) formed. In other words, at certain S_0,

$\frac{k_{pol} S_0}{K_d + S_0} = k_{off}$, and the fraction of 10-mer in the total products (sum of 9-mer and 10-mer) is 0.5.

During data analysis, the fraction of 10-mer was plotted against the concentration of ATP concentration, S, as shown in Figure S2, and the data was analyzed by data fitting to the following hyperbolic equation to derive, K_{app}

$$[10mer]_f = a\frac{S}{K_{app} + S} + b$$

Where b is the off-set accounting for the misincorporated fraction, a is the scaling factor, S is the concentration of the nucleotide under study. When $S = K_{app}$, fraction of 10-mer is 0.5, and thus from data fitting, K_{app} can be derived and it is the concentration S_0.

Now the K_{app} obtained from data fitting for different third nucleotide can be compared. Assuming influenza polymerase is similar to most polymerases, the rate of nucleotide incorporation during elongation, $k_{pol,}$ should be much faster than the rate of RNA release, $k_{off,}$ therefore, S_0 should be much smaller than K_d to satisfy this assumption.

As a result, $\frac{k_{pol} S_0}{K_d + S_0} = k_{off}$ can be reduced to $\frac{k_{pol} S_0}{K_d} = k_{off}$.

Since k_{off} is a constant, k_{pol}/K_d for each different nucleotide tested under the same conditions is <u>inversely proportional</u> to its S_0 or K_{app}. Here, k_{pol}/K_d is the specificity constant for nucleotide incorporation.

From K_{app}, the discrimination of the polymerase against different nucleotides can be calculated as

<u>Discrimination</u> = $\frac{k_{pol} / K_{d,correct}}{k_{pol} / K_{d,NTP}} = \frac{K_{app,NTP}}{K_{app,correct}}$

Similarly, the fidelity of the polymerase can be calculated as

<u>Fidelity (error rate)</u> = $\frac{k_{pol} / K_{d,NTP}}{k_{pol} / K_{d,NTP} + k_{pol} / K_{d,correct}} = \frac{K_{app,correct}}{K_{app,correct} + K_{app,NTP}}$
